# Myelodysplastic syndromes and idiopathic pulmonary fibrosis: a dangerous liaison

**DOI:** 10.1186/s12931-019-1151-6

**Published:** 2019-08-13

**Authors:** Spyros A. Papiris, Panagiotis Tsirigotis, Caroline Kannengiesser, Lykourgos Kolilekas, Konstantinos Gkirkas, Andriana I. Papaioannou, Patrick Revy, Paschalina Giouleka, Georgia Papadaki, Konstantinos Kagouridis, Vassiliki Pappa, Raphael Borie, Catherine Boileau, Demosthenes Bouros, Bruno Crestani, Effrosyni D. Manali

**Affiliations:** 1 2nd Pulmonary Medicine Department, General University Hospital “Attikon” Medical School, National and Kapodistrian University of Athens, 1 Rimini Street, 12462 Haidari, Athens, Greece; 2 2nd Department of Internal Medicine, Hematology Unit, General University Hospital “Attikon” Medical School, National and Kapodistrian University of Athens, Athens, Greece; 3 APHP Service de Génétique, Hôpital Bichat, Paris, France; 4 Université de Paris, Paris, France; 5 7th Pulmonary Department, Athens Chest Hospital “Sotiria”, Athens, Greece; 6 INSERM UMR 1163, Laboratory of GenomeDynamics in the Immune System, Imagine Institute, labéllisé Ligue contre le cancer, Paris, France; 7 APHP, Hôpital Bichat, Service de Pneumologie A, DHU FIRE Centre de référence des maladies pulmonaires rares, Paris, France; 8 Inserm U1152, Paris, France; 9 1st Department of Pneumonology, Athens Chest Hospital “Sotiria”, Athens, Medical School, National and Kapodistrian University of Athens, Athens, Greece

## Abstract

Previous studies have shown that the co-existence of bone marrow failure and pulmonary fibrosis in a single patient or in a family is suggestive of telomere related genes (TRG) germline mutations. This study presents the genetic background, clinical characteristics, and outcome of a group of five Greek patients co-affected with IPF and MDS. Four out of five patients developed an IPF acute exacerbation that was not reversible. We failed to detect any mutation in the *TERT, TERC, DKC1, TINF2, RTEL1, PARN, NAF1, ACD, NHP2* and *NOP10* genes in any patient. Moreover, telomere length was normal in the two patients tested. This could suggest that although the co-occurence of IPF and MDS are suggestive of TRG mutation in patients < 65 years old, in the elderly it may occur without germline mutations and could negatively affect prognosis. Physicians should be aware for possible IPF deterioration and therapeutic options for MDS should be wisely considered.

Both myelodysplastic syndromes (MDS) and idiopathic pulmonary fibrosis (IPF) constitute irreversible diseases of the elderly, and both share comorbidities that adversely affect their prognosis [1, 2]. Their co-occurrence exists and may relate to several factors including aging, but the impact of this type of liaison on each other’s prognosis has not yet received adequate attention [3, 4]. MDS presents with peripheral blood cytopenia, occasionally bone marrow fibrosis [1, 5] and encompasses a heterogeneous group of myeloid disorders that present increased risk to malignant transformation mainly to acute myeloid leukemia (AML) [1]. IPF is an irreversibly progressive fibrotic lung disorder [2]. Besides aging, the co-occurence of IPF and MDS has been reported in telomeropathies associated to telomeres related genes (TRG) [as telomerase reverse transcriptase (*TERT)* or telomerase RNA component (*TERC)*] germline mutations [6, 7] and short-telomeres [8]. Mutations in TRG are identified in 30% of patients with familial pulmonary fibrosis [9]. Furthermore, mutations in TRG have been shown to carry an unfavorable prognosis for IPF patients undergoing lung transplantation since TRG mutations increase the risk of severe bone marrow suppression and infections, eventually related to immunosuppression [7]. This study aims to present the genetic background, clinical characteristics, and outcome of a group of five consecutive Greek patients co-affected with IPF and MDS. All patients were diagnosed and followed-up at the Hematology and Pulmonary Medicine Department of a tertiary university hospital in Athens from March 2015 to March 2016 and all of them were included in the study due to clinical suspicion of telomeropathy based on the conjunction of pulmonary fibrosis and myelodysplasia [10]. The study has been approved by the decision No 937/22–7-15 of the “Attikon” hospital’s bioethics committee.

After written informed consent, all patients diagnosed with both IPF and MDS, underwent genetic testing for *TERT, TERC,* Dyskeratosis congenita 1 (*DKC1),* TERF1-interacting nuclear factor 2 *(TINF2),* Regulator of telomere length 1 *(RTEL1),* poly(A)-specific ribonuclease (*PARN),* Nuclear Assembly Factor 1 Ribonucleoprotein *(NAF1),* Adrenocortical dysplasia *(ACD),* H/ACA ribonucleoprotein complex subunit 2 *(NHP2)* and H/ACA ribonucleoprotein complex subunit 3 (*NOP10)* by next generation sequencing. Two of the five patients had their telomere lengths measured in peripheral blood cells by the telomeric restriction fragment (TRF) assay as already described [11]. IPF was diagnosed according to international consensus criteria [2] and MDS diagnosis was documented based on the World Health Organization 2016 classification criteria [5]. Autoimmune rheumatic diseases were excluded based on the absence of signs and symptoms of collagen vascular disease as well as on negative serological examination [2]. Patients’ data are reported in Table 1.

**Table 1:** Epidemiological, clinical, pulmonary function, hematology parameters, outcome and genetic background of 5 Greek patients with IPF and MDS

Parameters	#1	#2	#3	#4	#5
Gender	F	M	M	M	M
Age at IPF diagnosis (years)	74	82	74	78	84
Age at MDS diagnosis (years)	78	81	80	79	83
Age at death (years)	–	82	81	79	84
Duration of treatment with azacytidine (months)	0	14	9	1	0
Status	Alive	Dead	Dead	Dead	Dead
Time to death from diagnosis of IPF (months)	–	3	77	24	1
HRCT pattern	UIP	UIP	Probable UIP	Probable UIP	Probable UIP
Lung histology	–	–	UIP	–	UIP
Serology for CTD	negative	negative	negative	negative	negative
FVC % pred	88.6	–	80.8	70.5	68
DLCO % pred	59.2	–	58.3	77	65
GAP stage	I	III	I	II	II
WHO 2008 classification	RARS	RAEB II	RAEB I	RAEB I	RA
IPSS score	Low	Intermediate 2	Very high	Intermediate 1	Low
Treatment	–	Azacytidine	Azacytidine	Azacytidine	–
Cycles azacytidine	–	6	2	6	–
PO_2_/FiO_2_	357	118	137.5	142	73
Ht %	33.9	26.2	29.4	37.9	34.3
MCV (fL)	109.4	78.2	116.5	105.6	90.3
Neutrophils (G/L)	3210	600	540	1210	6700
PLT (G/L)	300	91	171	137	365
CRP (mg/L)	< 3	49.5	100	94.8	100
Family history	Yes^a^	No	No	No	No
*TRG* mutation	No	No	No	No	No
Telomere length (Kb)	10.9	NA	NA	NA	9.1

*IPF* idiopathic pulmonary fibrosis, *CTD* collagen vascular disease, *HRCT* high resolution computed tomography, *MDS* myelodysplastic syndrome; #: patient, *UIP* usual interstitial pneumonia, *RARS* refractory anemia ring sideroblasts, *RAEB* refractory anemia with excess blasts, *RA* refractory anemia, *FVC* forced vital capacity, *DLCO* diffusing capacity for carbon monoxide, *GAP* gender, age, lung physiology score, *PO*_*2*_*/FiO*_*2*_ ratio of arterial pressure of oxygen to fraction of inspired oxygen, *IPSS* international prognosis scoring system, *Ht* hematocrit, *WBC* white blood count, *MCV* mean corpuscular volume, *PLT* platelets, *CRP* C-reactive protein, *NA* not available, *TRG* telomere related gene. Family history: ^a^Mother died from liver cirrhosis. ‡ PO_2_/FiO_2_, Ht%, MCV, Neutrophils, PLT, Temperature, CRP regard the time point of the final respiratory event; the rest of the measurements regard baseline values

Five patients, with both IPF and MDS, four males, with a mean age of 80 (+/− 6) years, 60% ex- smokers were studied. Pulmonary function and hematology parameters are reported in Table 1. IPF diagnosis predated MDS diagnosis in three patients by 48, 77 and 18 months respectively. No patient received any anti-fibrotic or immunosuppressive agent. (Table 1). *TERT, TERC, DKC1, TINF2, RTEL1, PARN, NAF1, ACD, NHP2* and *NOP10* germline mutations were not detected in any patient. The telomere length was not pathologically reduced in the two patients tested (Table 1). MDS was classified as refractory anemia (RA) and RA with ring sideroblasts (RARS) in two patients both presenting low values for the international prognosis system score (IPSS). MDS was classified as RA with excess blasts (RAEB) in three patients presenting intermediate-1, intermediate-2 and high values for IPSS respectively (Table 1). RAEB patients were treated with azacytidine 75 mg/m^2^ subcutaneously for 7 days every 4 weeks [1, 5] and two of them developed acute leukemia. Four patients (including the three patients who received azacytidine) developed a fatal IPF acute exacerbation despite intensive supportive care, 12 (1–77) months post IPF diagnosis, 13 [9–14] months post MDS diagnosis and 9 [1–14] months post MDS treatment initiation with azacytidine.

Based on this case-series four out of five patients with the co-existence of IPF and MDS developed an IPF acute exacerbation that was not reversible. An extensive work-up was performed to exclude obvious causes of the deterioration such as infection, aspiration or drug toxicity [12]. Although azacytidine has been used safely in elderly patients and has been shown to significantly improve survival and quality of life [13], our observations suggest that this drug may not be as effective in patients with both IPF and MDS. Azacytidine is a hypomethylating agent and re-expression of tumor-suppressor genes has been suggested as a possible mechanism of action. However, hypomethylating activity is global and Azacytidine has a pleotropic effect on the immune system and could trigger the development of diffuse alveolar damage upon usual interstitial pneumonia through either an eventual toxicity of the drug to the lungs [1, 13] or infection because of the increased immunosuppression upon the vulnerable lungs of IPF patients [12, 14–16].

Despite previous studies showing that the co-existence of bone marrow failure and pulmonary fibrosis in a single patient or in a family is very suggestive of TRG germline mutations [6, 7], we failed to detect any mutation in the *TERT, TERC, DKC1, TINF2, RTEL1, PARN, NAF1, ACD, NHP2* and *NOP10* genes in any patient. Moreover, telomere length was normal in the two patients tested. In the study of Parry and co-workers [6] all patients were much younger compared to our patients with 6 out of 10 presenting with aplastic anemia in childhood or early adulthood and 4 out of them presenting with pulmonary fibrosis at an age younger than 61 years. Furthermore, all patients had a positive family history of either pulmonary fibrosis and MDS or aplastic anemia. The patients of the present study were much older and only one of them had a positive family history suggestive of short telomere syndrome.

Therefore, although the co-occurrence of IPF and MDS are suggestive of TRG mutation in patients < 65 years old, in the elderly it may develop without germline mutations or may relate to mutations in other genes that are still unknown. Both IPF and MDS are diseases of the elderly and may be triggered by physiologic aging processes affecting both the lungs and the bone marrow, such as stem cell exhaustion, mitochondrial dysfunction, epigenetic alterations and disturbances of DNA methylation [3, 4].

## Conclusion

This case series suggests that in the elderly patients the co-occurrence of IPF and MDS may develop without germline mutations and could negatively affect prognosis. Physicians should be aware for possible IPF deterioration and therapeutic options for MDS should be wisely considered.

## Data Availability

All data analyzed during this study are included in this published article.

## Abbreviations

*ACD*: Adrenocortical dysplasia
AML: Acute myeloid leukemia
CRP: C-reactive protein
*DKC1*: Dyskeratosis congenita 1
DLCO: Diffusing capacity for carbon monoxide
FVC: Forced vital capacity
GAP: Gender, age, lung physiology score
Ht: Hematocrit
IPF: Idiopathic pulmonary fibrosis
IPSS: International prognosis system score
MCV: Mean corpuscular volume
MDS: Myelodysplastic syndromes
NA: Not available
*NAF1*: Nuclear Assembly Factor 1 Ribonucleoprotein
*NHP2*: H/ACA ribonucleoprotein complex subunit 2
NOP*10*: H/ACA ribonucleoprotein complex subunit 3
*PARN*: Poly(A)-specific ribonuclease
PLTs: Platelets
PO_2_/FiO_2_: Ratio of arterial pressure of oxygen to fraction of inspired oxygen
RA: Refractory anemia
RAEB: Refractory anemia with excess blasts
RARS: Refractory anemia ring sideroblasts
*RTEL1*: Regulator of telomere length 1
*TERC*: Telomerase RNA component
*TERT*: Telomerase reverse transcriptase
*TINF2*: TERF1-interacting nuclear factor 2
TRG: Telomeres related genes
UIP: Usual interstitial pneumonia
WBC: White blood count
